# QTc prolongation is associated with severe desaturations in stroke patients with sleep apnea

**DOI:** 10.1186/s12890-022-01996-y

**Published:** 2022-05-24

**Authors:** Saara Sillanmäki, Jukka A. Lipponen, Henri Korkalainen, Antti Kulkas, Timo Leppänen, Sami Nikkonen, Juha Töyräs, Brett Duce, Aaron Suni, Samu Kainulainen

**Affiliations:** 1 Diagnostic Imaging Center, Kuopio University Hospital (KYS), P.O. Box 100, 70029 Kuopio, Finland; 2 Department of Applied Physics, University of Eastern Finland, Kuopio, Finland; 3 Department of Clinical Neurophysiology, Seinäjoki Central Hospital, Seinäjoki, Finland; 4 Science Service Center, Kuopio University Hospital, Kuopio, Finland; 5 The School of Information Technology and Electrical Engineering, The University of Queensland, Brisbane, Australia; 6 Department of Respiratory and Sleep Medicine, Sleep Disorders Centre, Princess Alexandra Hospital, Ipswich Rd, Woolloongabba, QLD Australia; 7 Institute for Health and Biomedical Innovation, Queensland University of Technology, Brisbane, Australia; 8 The School of Medicine, University of Eastern Finland, Kuopio, Finland

**Keywords:** QTc, Stroke, Obstructive sleep apnea, Desaturation, Repolarisation

## Abstract

**Background:**

Obstructive sleep apnea (OSA) is associated with vascular diseases from which stroke and sudden cardiac death are the most significant ones. It is known that disturbances of the autonomic nervous system and electrocardiographic changes are seen in patients with a previous cerebrovascular event. However, the pathophysiological cascade between breathing cessations, autonomic regulation, and cardiovascular events is not fully understood.

**Methods:**

We aimed to investigate the acute effect of desaturation on repolarisation in OSA patients with a previous stroke. We retrospectively analysed heart-rate corrected QT (QTc) intervals before, within, and after 975 desaturations in OSA patients with a stroke history and at least moderate sleep apnea (apnea–hypopnea index ≥ 15 events/h, *n* = 18). For the control population (*n* = 18), QTc intervals related to 1070 desaturation were analysed. Desaturations were assigned to groups according to their length and duration. Groupwise comparisons and regression analyses were further executed to investigate the influence of desaturation features on repolarization.

**Results:**

In the stroke population the QTc prolonged at least 11 ms during 27.1% of desaturations, and over 20 ms during 12.2% of desaturations. QTc was significantly prolonged during longer (> 30 s, *p* < 0.04) and deeper (> 7%, *p* < 0.03) desaturations. Less severe desaturations didn't influence QTc. In median, QTc prolonged 7.5 ms during > 45 s desaturations and 7.4 ms during > 9% deep desaturations. In the control population, QTc prolongation was observed but to a significantly lesser extent than in stroke patients. In addition, desaturation duration was found to be an independent predictor of QTc prolongation (β = 0.08, *p* < 0.001) among all study patients.

**Conclusions:**

We demonstrated that longer (> 30 s) and deeper (> 7%) desaturations prolong QTc in patients with stroke history. A significant proportion of desaturations produced clinically relevant QTc prolongation. As it is known that a long QTc interval is associated with lethal arrhythmias, this finding might in part explain the pathophysiological sequelae of cardiovascular mortality in OSA patients with a history of stroke.

## Introduction

Cardiovascular diseases are one of the leading causes of morbidity and mortality. Obstructive sleep apnea (OSA), a sleep disorder affecting approximately one billion adults worldwide [1], shares several common risk factors and comorbid conditions with cardiovascular disease. Furthermore, OSA is associated with an increased risk of cerebrovascular stroke [2]. It has been shown that 90% of patients with a previous stroke have at least mild OSA (apnea–hypopnea index (AHI) ≥ 5 events/h) [3]. This connection between stroke, OSA, and cardiac arrhythmogenicity is clinically relevant since OSA is a known risk factor for sudden cardiac death (SCD) [4].

Cerebrovascular stroke can cause changes in autonomic tone, electrocardiographic abnormalities, and increase the incidence of lethal cardiac arrhythmias [5]. Furthermore, it has been shown that stroke survivors with long QT intervals are at a higher risk of cardiac death [6]. In most cases, sudden, unexpected death is caused by fatal cardiac arrhythmias [7] while QT prolongation is a risk factor for the development of ventricular tachycardia and even SCD [8]. Although extensive research has been carried out on electrocardiogram (ECG) changes and arrhythmia in OSA patients, there are no studies adequately investigating QT interval prolongation relative to desaturation features in patients with known stroke history.

Even though the AHI is the most used parameter for OSA severity estimation, it fails as a benchmark for detailing the OSA severity. Thus, parameters describing physiological cardiopulmonary consequences are needed [5–7]. Moreover, a greater understanding of the connection between respiratory events (breathing cessations and obstructions) and cardiovascular electrophysiological alterations may provide useful clinical insights into the causes and prevention of SCD. Therefore, we investigated this association in a population comprising OSA patients having suffered a stroke. Our main hypothesis was that deeper and longer desaturation cause greater prolongation of the QTc interval in OSA patients with a previous stroke. We aimed to study whether OSA-related desaturations are associated with repolarization prolongation and to investigate which desaturation features (e.g., length or depth) causes the highest risk for QTc prolongation predisposing to a possible risk for ventricular arrhythmias.

## Methods

### Study population

The initial dataset used in this study comprised (*n* = 916) suspected OSA patients, who had undergone type I full diagnostic polysomnography (PSG) at the Princess Alexandra Hospital (Brisbane, Australia) during 2015–2017. The PSGs were performed by using the Compumedics Grael acquisition system (Compumedics, Abbotsford, Australia). From the initial dataset, patients with a previous stroke and controls were matched based on OSA severity.

Approval for retrospective data collection was obtained from the Institutional Human Research Ethics Committee of the Princess Alexandra Hospital (HREC/16/QPAH/021 and LNR/2019/QMS/54313). All procedures were performed following the ethical standards of the institutional and national research committee, and in accordance with the Helsinki declaration. The need for informed consent was waived by the Metro South Human Research Ethics Committee due to the retrospective nature of the study.

Out of the initial data set, 21 subjects fulfilled the inclusion criteria for this study: previous cerebrovascular stroke and at least moderate sleep apnea (AHI ≥ 15 events/h) with a total sleep time of ≥ 4 h in the PSG. Within these patients, the quality of ECG was insufficient in two cases and one patient was excluded due to ventricular pacing. No other exclusion criteria were used. Finally, eighteen patients were included in the further analyses. The reference population (*n* = 18) was matched based on OSA severity from the same dataset with the same inclusion criteria but excluding patients with a history of stroke. Demographic information for the stroke and control population are presented in Tables 1 and 2, respectively.

**Table 1:** Demographic, PSG, and ECG characteristics in the stroke population

Demographics	*n/*Median (%/IQR)	ECG characteristics	Median (IQR)
Patients (male)	18 (66.7)	RR interval (ms)	923.9 (834.1–1060.2)
Age (years)	67.9 (55.9–71.9)	PQ interval (ms)	188.9 (174.0–212.6)
BMI (kg/m^2^)	35.8 (33.7–41.8)	QRS interval (ms)	94.0 (85.3–102.0)
Atrial fibrillation	1 (5.6)	QT interval (ms)	417.9 (384.0–452.8)
COPD	1 (5.6)	QTc interval (ms)	424.1 (409.7–455.3)
Dyslipidemia	8 (44.4)	**Nocturnal HVR**	**Median (IQR)**
Hypertension	13 (72.2)	SDNN (ms)	33.7 (16.0–41.2)
Smoker	2 (11.1)	RMS-SD (ms)	21.7 (14.1–47.4)
**PSG characteristics**	**Median (IQR)**	LF (m^2^)	404.4 (134.9–710.6)
AHI (events/h)	35.3 (23.5–45.5)	HF (m^2^)	175.6 (43.3–519.5)
ODI (events/h)	23.1 (14.9–37.1)	LF/HF	1.4 (0.6–3.4)
Desaturation duration (s)	28.0 (19.6–41.0)	VLF (m^2^)	188.8 (56.8–334.9)
Desaturation depth (%)	4.3 (3.1–6.5)	SD1 (ms)	15.4 (10.0–33.5)
TST (min)	319.0 (285.3–351.9)	SD2 (ms)	40.8 (20.7–49.4)
Arousal index (events/h)	32.3 (24.1–44.1)	SD1/SD2	1.7 (1.0–2.9)

*PSG* polysomnography, *ECG* electrocardiogram, *n* number of patients, *BMI* body mass index, *COPD* chronic obstructive pulmonary disease, *IQR* interquartile range, *AHI* apnea–hypopnea index, *ODI* oxygen desaturation index, *TST* total sleep time, *HRV* heart rate variability, *SDNN* standard deviation of NN intervals, *RMS-SD* root mean square of successive RR interval differences, *LF* absolute power of the low-frequency band, *HF* absolute power of the high-frequency band, *LF/RF* ratio of LF-to-HF power, *VLF* absolute power of the very-low-frequency band, *SD1* Poincaré plot standard deviation perpendicular the line of identity, *SD2* Poincaré plot standard deviation along the line of identity, *SD1/SD2* ratio of SD1-to-SD2

**Table 2:** Demographic, PSG, and ECG characteristics in the reference population

Demographics	*n/*Median (%/IQR)	ECG characteristics	Median (IQR)
Patients (male)	18 (72.2)	RR interval (ms)	887.1 (808.1–998.3)
Age (years)	48.3 (46.2–49.6)	PQ interval (ms)	179.7 (164.1–191.6)
BMI (kg/m^2^)	35.1 (31.0–37.9)	QRS interval (ms)	93.8 (85.0–102.1)
Atrial fibrillation	1 (5.6)	QT interval (ms)	390.6 (359.4–410.2)
COPD	0 (0)	QTc interval (ms)	405.0 (392.7–420.8)
Dyslipidemia	6 (33.3)	**Nocturnal HVR**	**Median (IQR)**
Hypertension	7 (38.9)	SDNN (ms)	38.2 (17.7–54.2)
Smoker	3 (16.7)	RMS-SD (ma)	29.7 (14.7–52.5)
**PSG characteristics**	**Median (IQR)**	LF (m2)	563.2 (180.6–1456.1)
AHI (events/h)	29.8 (24.3–37.8)	HF (m2)	320.5 (79.1–788.4)
ODI (events/h)	25.1 (13.2–40.7)	LF/HF	2.1 (1.3–3.6)
Desaturation duration (s)	30.0 (21.1–40.0)	VLF (m^2^)	186.7 (91.9–481.5)
Desaturation depth (%)	4.7 (3.4–7.9)	SD1 (ms)	21.9 (10.4–37.2)
TST (min)	339.8 (321.0–368.5)	SD2 (ms)	44.5 (23.7–67.7)
Arousal index (events/h)	28.5 (23.9–41.2)	SD1/SD2	2.1 (1.8–2.7)

*PSG* polysomnography, *ECG* electrocardiogram, *n* number of patients, *BMI* body mass index, *COPD* chronic obstructive pulmonary disease, *IQR* interquartile range, *AHI* apnea–hypopnea index, *ODI* oxygen desaturation index, *TST* total sleep time, *HRV* heart rate variability, *SDNN* standard deviation of NN intervals, *RMS-SD* root mean square of successive RR interval differences, *LF* absolute power of the low-frequency band, *HF* absolute power of the high-frequency band, *LF/RF* ratio of LF-to-HF power, *VLF* absolute power of the very-low-frequency band, *SD1* Poincaré plot standard deviation perpendicular the line of identity, *SD2* Poincaré plot standard deviation along the line of identity, *SD1/SD2* ratio of SD1-to-SD2

### PSG analysis

The PSG recordings were manually scored according to the American Academy of Sleep Medicine 2012 guidelines [9] by experienced sleep technicians in the Princess Alexandra Hospital using Compumedics ProFusion PSG 4 software (Compumedics, Abbotsford, Australia). All ≥ 3% desaturations were scored from the onset of the desaturation to the recovery of oxygenation and all desaturations during wake were excluded. The scoring process is described in a previous paper [10]. The detailed information of each desaturation was exported from ProFusion to MATLAB (R2019b; Mathworks, Natick, MA, USA) for data processing.

### ECG analysis related to desaturation events

**Fig. 1:**
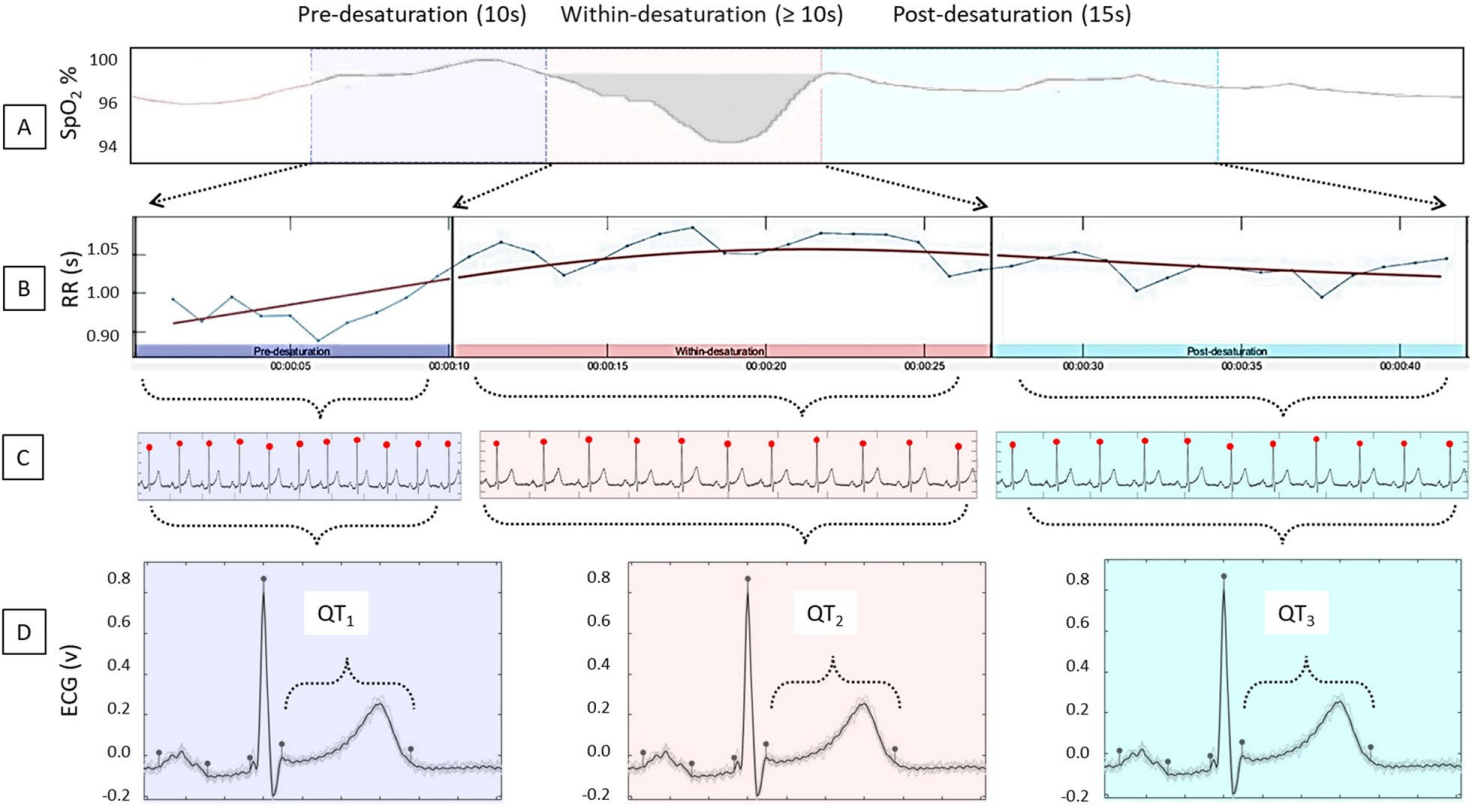
At first, the desaturation was detected, and the electrocardiography (ECG) signal samples were extracted based on the start and end times of each desaturation (**A**). The ECG signal was examined in three parts: 10-s pre-desaturation ECG sample, ECG sample within-desaturation, and 15-s post-desaturation ECG sample (**B**). The R-wave fiducial points were detected (**C**) and an average waveform for electrical activity was computed for each ECG sample in all three parts (**D**). The average QT times are measured for 10 s pre desaturation (QT_1_), within-desaturation (QT_2_), and 15 post-desaturation (QT_3_)

ECG signal samples were extracted based on the start and end times of each desaturation. The nocturnal ECGs (lead II) were recorded during the PSG study with a sampling frequency of 256 Hz. The ECG signal was examined in three parts: (1) 10-s pre-desaturation ECG sample before the onset of the desaturation, (2) ECG sample within-desaturation, and (3) 15-s post-desaturation ECG sample after the end of desaturation (Fig. 1). If the post- and pre-desaturation ECG samples of two consecutive desaturations overlapped, all samples of the latter desaturation were excluded from further analyses. In addition, desaturations with a duration of less than 10 s were excluded. After these exclusions, a total of 975 (out of 2310) ECG samples were included in further analyses for the stroke population. For the reference population (*n* = 18), a total of 1070 (out of 3277) ECG samples were included in the analyses.

After extracting the ECG samples, the R-wave fiducial points were detected using Kubios HRV Premium software (Kubios Oy, Kuopio, Finland) [11] and detections were verified by visual inspection of all extracted ECG samples (Fig. 1). Ventricular extrasystoles were excluded from the analysis. The average waveform for electrical activity was computed for each ECG sample in all three categories (i.e. before, within, and after the related desaturation) to produce averaged P-QRS-T segments. The onset and offset times of the averaged P-, QRS-, and T-waves were manually set for each sample using Kubios HRV software [11] by trained ECG readers (Fig. 1). Heart rate corrected QT (QTc) intervals were calculated using Bazett’s formula [12].

The nocturnal heart rate variability (HRV) parameters were analysed with Kubios Premium software. The ECG samples 30-min after the onset of PSG recording and 30-min before the end of PSG recordings were excluded from the nocturnal HRV analysis. The average of the analysed HRV recording was 6 h.

To investigate the effect of desaturation characteristics on QTc intervals, the desaturations were first grouped based on their duration (10 ≤ s < 20, 20 ≤ s < 30, 30 ≤ s < 45, and ≥ 45 s) and depth, i.e. the magnitude of blood oxygen saturation (SpO_2_) drop (3% ≤ **Δ**SpO_2_ < 5%, 5% ≤ **Δ**SpO_2_ < 7%, 7% ≤ **Δ**SpO_2_ < 9%, and **Δ**SpO_2_ ≥ 9%).

### Statistical analysis

The statistical significance of the differences in QTc intervals between different duration and depth groups was evaluated using Wilcoxon’s rank-sum (W) test. Additionally, the statistical significance of differences between pre-desaturation QTc intervals and within-desaturation QTc intervals was evaluated using Wilcoxon’s signed-rank sum (T) test as the within-desaturation QTc was assumed to be dependent on the pre-desaturation QTc time. The limit for statistical significance was set to be *p* < 0.05. The statistical significance of differences between the reference population and the stroke population were evaluated with Wilcoxon’s W test (baseline characteristics) and the two-sided Kolmogorov–Smirnov test (distributions of ΔQTc). Moreover, generalized linear regression and stepwise linear regression were conducted to analyze the effect of demographics, conventional OSA severity, and event-by-event desaturation characteristics on the ΔQTc between pre- and within-desaturation segments. In stepwise regression, deviance with *p*-value’s F-statistics was used as entering and removing criteria.

## Results

QTc was prolonged (at least 1 ms) during 52.6% of desaturations in the stroke population. Moreover, QTc was prolonged for at least 11 ms during 27.1% of desaturations and over 20 ms during 12.2% of desaturation (Table 3). In the reference population, most of the prolongations were minor (1–11 ms, 38.2%), and only 4.0% of the desaturations caused over 20 ms prolongation (Table 3).

**Table 3:** The effect of desaturation characteristics on QTc intervals in the stroke and control population

ΔQTc	Number of desaturations	Desaturation duration (s)	Desaturation depth (%)
*Stroke population*			
1–11 ms	264 (27.1%)	28.2 (19.8–40.0)	3.9 (3.0–7.4)
11–16 ms	82 (8.4%)	40.0 (26.0–49.0)	5.0 (3.4–11.2)
16–20 ms	48 (4.9%)	30.5 (22.5–44.0)	4.1 (3.2–7.2)
> 20 ms	119 (12.2%)	30.0 (18.1–43.8)	4.7 (3.4–7.3)
*Control population*			
1–11 ms	409 (38.2%)	30.0 (22.0–40.0)	4.6 (3.3–7.2)
11–16 ms	57 (5.3%)	28.0 (18.0–40.5)	4.3 (3.2–7.3)
16–20 ms	37 (3.5%)	34.0 (22.9–42.8)	4.7 (3.9–6.2)
> 20 ms	43 (4.0%)	34.0 (18.3–43.8)	5.2 (3.7–7.0)

Desaturations are grouped according to the absolute change in the heart rate corrected QT interval (ΔQTc) within desaturations. The median and (interquartile range) of desaturation duration (s) and depth (%) are presented related to each ΔQTc group

**Fig. 2:**
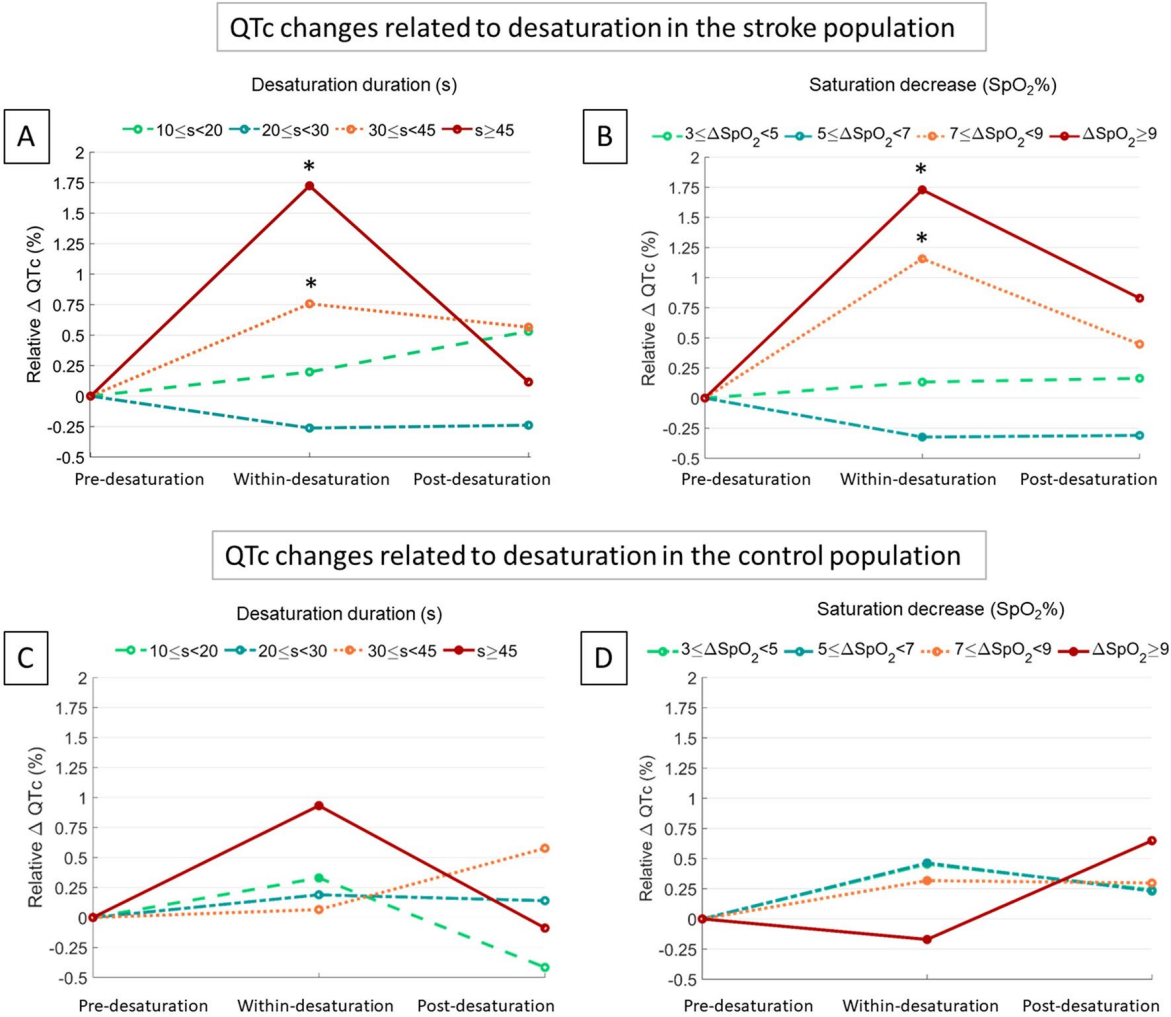
The median relative changes in QTc (∆QTc) interval (within- and post-desaturation) compared to the median pre-desaturation QTc levels both in the stroke population (*n* = 18, subfigures **A** and **B**) and in the control population (*n* = 18, subfigures **C** and **D**). Changes were categorized by the length (**A** and **C**) and by the dept of the desaturation (**B** and **D**). The number of desaturations in each desaturation group varied from 79 to 571. *Statistically significant (*p* < 0.05) difference in ∆QTc between stroke and control population

To further study the influence of desaturation characteristics on QTc among stroke patients, desaturations were also categorized into sub-groups according to the desaturation duration (Table 4; Fig. 2). The pre-desaturation QTc was longer before 30 ≤ s < 45 and ≥ 45 s desaturations compared to short (10 ≤ s < 20) desaturations (*p* < 0.01 for both) (Table 4). There was no statistical significance in pre-desaturation QTc durations between the other desaturation duration groups. Furthermore, the QTc interval within-desaturations was significantly (*p* < 0.01) longer compared to the corresponding pre-desaturation QTc intervals in the 30 ≤ s < 45 and ≥ 45 s desaturation duration groups (Table 4; Fig. 2A). In shorter desaturation duration groups, QTc intervals did not change significantly (*p* > 0.11) within-desaturations compared to pre-desaturation QTc intervals. The median QTc was slightly longer after 10 ≤ s < 20 and 30 ≤ s < 45 desaturations compared to corresponding pre-desaturation QTc intervals (2.3 and 2.4 ms, *p* < 0.05 for both). There were no statistically significant differences in pre-desaturation and post-desaturation QTc intervals in 20 ≤ s < 30 and ≥ 45 s desaturation duration groups (Table 4). No statistically significant difference between pre-desaturation and post-desaturation QTc in the stroke population (Table 4). In the reference population, only mild prolongation was seen post-desaturation after 10 ≤ s < 20 (median 2.3 ms) and 30 ≤ s < 45 (median 2.4 ms) (Table 4). In the reference population pre-desaturation QTc was longer before 10 ≤ s < 20 compared to ≥ 30 s desaturations (p < 0.05). Only the ≥ 45 s desaturations caused significant (*p* < 0.01) prolongation of the QTc among the reference population. Furthermore, in 30 ≤ s < 45 and ≥ 45 s desaturation duration groups, stroke patients had a significantly (*p* < 0.04) greater change between pre-desaturation and within-desaturation QTc intervals compared to the reference population (Fig. 2A, C).

**Table 4:** Median (interquartile range) changes in QTc in the stroke and control population

	Pre-desaturation QTc (ms)	ΔQTc within-desaturation (ms)	ΔQTc post-desaturation (ms)
Desaturation duration	Stroke population		
10 ≤ s < 20	418.9 (405.4–439.4)	0.8 (− 8.9–9.7)	**2.3 (**− **7.4**–**11.0)**
20 ≤ s < 30	422.3 (409.5–453.5)	− 1.1 (− 8.0–8.7)	− 1.0 (− 11.3–7.8)
30 ≤ s < 45	427.4 (412.7–457.2)^*^	**3.4 (**− **6.9**–**12.3)**	**2.4 (**− **6.3**–**10.5)**
≥ 45 s	430.6 (412.6–460.9)^*^	**7.5 (**− **3.0**–14.8) *	0.5 (− 10.3–10.8)
Desaturation depth			
3% ≤ ΔSpO2 < 5%	424.8 (409.1–454.9)	0.6 (− 8.9–11.1)	0.8 (− 11.5–9.5)
5% ≤ ΔSpO2 < 7%	424.8 (414.1–458.3)	− 1.4 (− 9.7–10.5)	− 1.4 (− 9.8–9.4)
7% ≤ ΔSpO2 < 9%	424.5 (408.9–444.1)	**4.6 (**− **3.2**–12.3)^*^	2.0 (− 4.8–7.8)^*^
ΔSpO2 ≥ 9%	424.3 (413.7–437.4)	**7.4 (**− **1.1**–14.9)^*^	**3.7 (**− **5.6**–**10.0)**^†^
Desaturation duration	Control population		
10 ≤ s < 20	416.2 (390.9–436.7)	1.4 (− 5.2–8.1)	− 1.7 (− 7.9–5.9)
20 ≤ s < 30	405.8 (392.1–424.9)	0.8 (− 5.1–6.4)	0.6 (− 6.2–7.6)
30 ≤ s < 45	401.5 (391.2–413.4)^*^	0.3 (− 4.5–5.6)	2.2 (− 3.6–10.1)^*^
≥ 45 s	403.2 (391.3–414.5)^*^	**3.7 (**− **2.6**–**8.9)**^**†**^	− 0.4 (− 4.9–6.2)
Desaturation depth			
3% ≤ DSpO2 < 5%	402.6 (387.0–423.1)	**1.8 (**− **3.7**–**6.8)**	0.9 (− 4.7–7.9)
5% ≤ SpO2 < 7%	405.0 (390.8–422.0)	**1.8 (**− **3.5**–**8.2)**	0.8 (− 5.8–9.5)
7% ≤ SpO2 < 9%	405.5 (397.3–421.4)	1.3 (− 6.5–6.1)	1.2 (− 7.8–6.7)
SpO2 ≥ 9%	404.2 (396.3–414.9)	− 0.7 (− 7.3–5.1)^*^	2.6 (− 4.9–9.6)

Change in the within-desaturation segment (ΔQTc within-desaturation) and the post-desaturation segment (ΔQTc post-desaturation) were calculated based to the pre-desaturation QTc segment values. Bolded values indicate a statistically significant (*p* < 0.05) change from the pre-desaturation QTc value. ^*^The value differs significantly from the 10–20 s or 3–5% group. ^†^The value differs significantly from all duration or depth groups. Abbreviations: **Δ**SpO2 = drop in the blood oxygen saturation during desaturation

We also categorized desaturations into sub-groups according to the depth of desaturation. There were no statistically significant differences in pre-desaturation QTc intervals in the stroke group (Table 4). However, the QTc prolonged significantly during 7% ≤ **Δ**SpO_2_ < 9% and ≥ 9% desaturations (Table 4; Fig. 2B). QTc did not prolong significantly during < 7% desaturations. Compared to pre-desaturation QTc, the post-desaturation QTc was slightly elevated (median 3.7 ms) after ≥ 9% desaturations in stroke patients. No statistically significant difference between pre-desaturation and post-desaturation QTc intervals was observed in other desaturation depth groups or in the reference population (Table 4). In the reference population, the QTc prolongations were minor, and the deeper desaturations did not similarly affect the QTc than in stroke patients (Table 4). Moreover, the deep (ΔSpO_2_ ≥ 7%) desaturations caused significantly (*p* < 0.01) greater QTc prolongation in the stroke patients compared to the reference population.

In regression analysis, both in the general linear (GL) model as well as in the stepwise (SW) model, only the total sleep time in PSG (β_GL_ =   .366, β_SW_ = −1.549, *p* < 0.01) and desaturation duration (β_GL_ = 0.065, β_SW_ = 0.080, *p* < 0.002) were significant predictors of the increase in ∆QTc (Table 5). Conversely, age, sex, or AHI were not predictors of acute QTc prolongation.

**Table 5:** Generalized linear regression model and a stepwise regression model for absolute ∆QTc (ms) between within-desaturation period and pre-desaturation in stroke and control population

Generalized model	β	SE	T	*p*
Sex	0.578	0.774	0.747	0.455
Age (y)	0.049	0.037	1.310	0.190
TST (h)	− **1.366**	**0.524**	− **2.591**	**0.010**
AHI (1/h)	− 0.031	0.018	− 1.725	0.09
Desaturation duration (s)	**0.065**	**0.022**	**2.978**	**0.002**
∆SpO_2_ (%)	0.069	0.077	0.895	0.371

Stepwise model	β	SE	T	*p*
Sex	–	–	–	–
Age (y)	–	–	–	–
TST (h)	− **1.549**	**0.457**	− **3.391**	** < 0.001**
AHI (1/h)	–	–	–	–
Desaturation duration (s)	**0.080**	**0.021**	**3.818**	** < 0.001**
∆SpO_2_ (%)	–	–	–	–

All other parameters except Desaturation duration and ∆SpO_2_ are treated patient-wise; the two are computed for each segment separately. The criteria for excluding parameters from the stepwise model was Deviance and *p*-value for F-statistics. Abbreviations: *TST* total sleep time, *AHI* apnea–hypopnea index, ∆SpO_2_ = depth of desaturation

## Discussion

In the present study, we evaluated the acute influence of desaturation length and depth in repolarization duration in OSA patients with a previously diagnosed stroke, and OSA severity matched counterparts without a previous stroke. We found that longer and deeper desaturations can prolong QTc interval during a desaturation, especially in patients with previous stroke. Moreover, the desaturation length was an independent predictor for QTc prolongation in both stroke and control populations.

Intermittent hypoxemia is assumed to promote cardiac arrhythmias by producing oxidative stress, systemic inflammation, and increased sympathetic activity leading to anatomical and electrophysiological remodeling [13, 14]. Interestingly, it has been shown that hypoxia induces QTc prolongation also in healthy subjects [15]. Still, the pathophysiological cascade between breathing cessations, autonomic dysregulation, and fatal cardiovascular events is partly unclear. Based on previous studies, we hypothesized that desaturation leads to acute intermittent prolongation of the QTc. Since the autonomic nervous system (ANS) plays an important role in the regulation of repolarization [16] we focused on OSA patients with a previous stroke as stroke can lead to impaired autonomic control [19]. As hypothesized, we found that as the heart rate increases within-desaturations the shortening of the QTc interval was inadequate among OSA patients with a previous stroke. We further evaluated ANS activation in our patient populations with nocturnal HRV. The stroke patient had slightly lower median HRV parameters compared to the control population, which can suggest that they tend to have lower ANS function.

OSA causes acute and long-time sympathetic autonomic nervous system activation by increasing pulmonary artery pressure and chemoreceptor activation caused by acidosis, hypoxia, and hypercapnia [17]. Previously, Tavares et al. have shown that when blood SaO_2_ levels decline, the sympathetic activation increases progressively [17]. Moreover, a cohort study showed that the lowest nocturnal O_2_ saturation value and the mean O_2_ saturation at night, are independent predictors of SCD [4]. These findings are in line with our results since we found that more substantial desaturations are associated with QTc prolongation which is a risk factor for lethal arrhythmias [8]. The ANS activation alters not only heart rate, conduction, and hemodynamics, but also the cellular and subcellular properties of individual myocytes [18]. A previous study has shown that intrathoracic pressure swings cause acute left-sided heart morphology and function changes [19] and this may lead to electrophysiological remodeling and further to the manifestation of arrhythmias.

The QTc prolongation seen in the present study might explain the increased risk for nocturnal SCD of OSA patients with long desaturations for the following reasons. First, it has been shown that the frequency and duration of respiratory and desaturation events increase towards the morning [20]. Second, it is known that both prolonged QTc and dynamic changes in QT interval increase the risk for arrhythmias [21]. Third, it has been shown that the risk for sudden death from cardiac causes is increased in OSA patients from midnight to 6 a.m. [22]. These previous findings reinforce the association between pro-arrhythmogenic QTc prolongation and long (> 30 s) and deep (> 7%) desaturations.

The retrospective nature of the study leads to certain limitations. First, we had no information available on the anatomical location of the stroke or the time between stroke and PSG/ECG recordings. Thus, there is the possibility that autonomic regulation may improve during the intervening period between the stroke event and the diagnostic PSG. However, a previous study has shown that the long-term negative effects on cardiovascular regulation can still be seen after stroke [23]. Secondly, the stroke patients were older compared to controls, the difference in acute electrophysiological response on desaturation may be also influenced by longer exposure to the pathological insults associated with sleep apnea and other comorbidities. However, the results of regression analyses showed that when sex, age, and conventional OSA severity are considered alongside desaturation metrics, desaturation duration was the only independent predictor of acute QTc prolongation together with total sleep time (Table 5). Therefore, even though the stroke population was older, and had higher baseline QTc, the acute effect of desaturation to repolarization is significant and modulated especially by the length of the desaturation event. Thus, the difference in QTc prolongation between the populations cannot be explained by age or AHI. Yet, we know that TST is related to age so the age might not be completely irrelevant and further analyses outside the scope of this study are needed.

Moreover, we had to assume an appropriate evaluation window for ECG samples before and after desaturation. For this reason, we excluded desaturations originating less than 25 s after the previous desaturation had ended. It is possible that the exclusion of a significant number of desaturations can cause some bias in the result and can partly explain why short (10 ≤ s < 20) desaturations seem to cause a more substantial influence on QTc than longer (20 ≤ s < 30) desaturations (Fig. 2A). Furthermore, it is common that patients with very severe sleep apnea suffer from frequent desaturations that are short but cause rapid drops in SpO_2_ level and can still be very deep. Therefore, the desaturations of these patients would be mostly represented in the short (10 ≤ s < 20) desaturation group. This can also partly explain why short desaturations seem to have a greater effect on QTc interval. Another reason behind this unexpected finding can be due to desaturations being estimated from a level just before desaturation onset and wake-time normal blood saturation levels are not considered. It is possible that in these measurements, the short events start during already lower SpO_2_ levels, causing SpO_2_ to drop faster and further induce QTc prolongation. Last, the QT measuring and correction can be challenging. In this study, the QTc was measured semiautomatically from the lead II which is the most often used in QT interval duration measurements because it is more likely to have the longest QT interval [24]. In addition, several approaches have been developed to correct the QT interval. It is known that the Bazett's correction formula is not optimal due to its tendency to underestimate and overestimate the duration of cardiac repolarization at extremely low and high heart rates. However, we chose to use the Bazett´s formula as it is the most used and universal QT interval correction method to achieve results that are easily comparable with the literature. The median heart rate in this study was 64.9 beats/min in the stroke population and 67.6 beats/min in the reference population (Tables 1, 2). Thus, as there were no major differences in median heart rates, we consider that using the Bazett’s formula does not have major effects on the obtained results.

## Clinical implications and conclusions

In the stroke population, 12.2% of desaturations induced ≥ 20 ms prolongation in QTc (Table 3). Generally, when drug safety is estimated, prolongation of QTc of ≥ 20 ms is associated with a substantial likelihood of the drug being proarrhythmic [25]. Therefore, we consider that OSA should be recognized as a risk factor for the acquired long-QT syndrome (LQTS) in stroke patients. Moreover, when describing medication associated with QT prolongation possible nocturnal QTc prolongation should be noticed. We believe that this is essential, especially in OSA patients diagnosed with LQTS or if they have borderline daytime QT levels.

In the current clinical practice, atrial fibrillation is routinely screened with 24-h Holter monitoring if no obvious cause of stroke can be found. However, routine screening for sleep-disordered breathing is uncommon in patients with stroke even though OSA is overrepresented in this patient group [3]. Combining home polysomnography with the 24-h Holter monitoring would be a feasible and possibly beneficial addition to stroke patients’ clinical risk evaluation. This is because sleep apnea is an important target for public health interventions aiming at reducing the risk for arrhythmias and SCD. Moreover, continuous positive airway pressure treatment has been shown to significantly decrease QTc in moderate/severe OSA patients [26]. Our finding might partially explain why continuous positive airway pressure therapy, which decreases the frequency and duration of desaturations, prevents cardiac arrhythmias [13]. This finding endorses the need to screen and treat OSA, especially in patients with a previous stroke.

## Data Availability

The datasets analysed during the current study are not publicly available due to privacy policy but are available from the corresponding author on reasonable request.
